# Does tail docking prevent *Cochliomyia hominivorax* myiasis in sheep? A six-year retrospective cohort study

**DOI:** 10.1017/awf.2024.21

**Published:** 2024-05-08

**Authors:** Giuliano Pereira de Barros, Maria José Hötzel, Marceli Carvalho da Silva, Laura Lívia Arias Avilés, Patrizia Ana Bricarello

**Affiliations:** Departamento de Zootecnia e Desenvolvimento Rural, Universidade Federal de Santa Catarina, Florianópolis 88034-001, Brazil

**Keywords:** Animal science, animal welfare, blowfly, flystrike, parasitology, veterinary epidemiology

## Abstract

Tail docking is a husbandry practice widely incorporated in sheep farms around the world. It is an irreversible mutilation that impairs animal welfare, both immediately and in the longer term. The defence of tail docking as a practice is centred around the perception that doing so contributes to the promotion of local hygiene, allowing the use of the wool, facilitating reproductive management and reducing the chances of myiasis, a disease caused by the invasion of blowfly larvae in the tissues of warm-blooded animals. However, current understanding of farm animal welfare questions the need to maintain practices such as tail docking. Thus, the aim of this study was to evaluate the effect of tail docking on the incidence of *Cochliomyia hominivorax* myiasis in sheep in an experimental flock in Brazil during a six-year retrospective cohort study. Relative risk, odds ratio and incidence rate ratio were the association measures adopted. A total of 4,318 data-points were collected and supplied the analytical model. Tail docking did not decrease the risk and, on the contrary, was found to increase the chances of sheep being affected by myiasis. The results support the hypothesis that tail docking is not a protective factor against the occurrence of myiasis and further fuel calls for a rethink of tail docking being deployed as a blanket measure in the prevention of myiasis in sheep.

## Introduction

Tail docking, or caudectomy, is one of the most prevalent types of mutilation, intentionally carried out on domestic animals worldwide (Sutherland & Tucker 2011). Domestic animals of different species undergo this management for different reasons. With regard to farm animals, caudectomy is viewed as a ‘husbandry practice’ and is commonly deployed, especially in pigs and sheep, but also in dairy cattle (Sutherland & Tucker 2011).

Tail docking is seen in the vast majority of sheep flocks throughout the world, and is perceived as a practice inherent to sheep farming that confers benefits to the health of the animals (Larrondo *et al.* 2018; Stamm *et al*. 2019; Woodruff *et al.* 2020). In the UK it is common to dock sheep within the first week of life, but in more extensive systems, such as those used in New Zealand and Australia, animals are likely to be older. Tail docking is performed without the use of any type of analgesia or anaesthesia for animals under eight days of age (Sutherland & Tucker 2011). Even recognising that sheep have the capacity to feel pain and experience suffering, the vast majority of the stakeholders involved, including sheep farmers, do not express a desire to abolish it (Larrondo *et al.* 2018; Stamm *et al*. 2019; Woodruff *et al.* 2020).

Dialogue regarding the maintenance of this practice is emerging in Brazil and other parts of the world (Sheep Standards and Guidelines 2013; Soriano *et al.* 2020), originating mainly from outside the livestock sector, among consumers (Alonso *et al.* 2020; Connor & Cowan 2020). This represents part of a societal shift in attitude that derives from differences in the perception of the welfare of farmed animals (Vargas-Bello-Pérez *et al.* 2017). Husbandry practices that generate pain and suffering in farm animals are being increasingly questioned by the general public (Hötzel & Vandresen 2022), and the debate has entered into the universities (Orihuela & Ungerfeld 2019).

In Brazil a number of legal measures and recommendations of official normative bodies related to avoidance of suffering arising from tail docking are in place (Soriano *et al.* 2021). For example, the Brazilian legislation for the certification of organic systems of sheep production does not permit the practice of tail docking (Ministério da Agricultura, Pecuária e Abastecimento 2021). However, this discussion is still ongoing. The Normative Resolution nº 877 of February 15, 2008 of the Brazilian Federal Veterinary Council, in Article nº 5, paragraph 2, defined tail docking in sheep as a prohibited practice (Conselho Federal de Medicina Veterinária 2008). This decision met significant resistance from farmers and was subsequently modified through Normative Resolution No 982 of November 13, 2009 of the same council, which stipulated in Article No 6 that tail docking was allowed in woolly sheep breeds. Wool sheep have a greater concentration in southern Brazil, Minas Gerais, São Paulo and Mato Grosso do Sul States, while flocks of hairy breeds make up the largest contingent of sheep in north-eastern Brazil (Instituto Brasileiro de Geografia e Estatística 2021). Annex 2 of this same Normative Resolution declares the practice may only be performed if the animals have undergone anaesthesia and analgesia (Conselho Federal de Medicina Veterinária 2009). In England and Wales, tail docking of sheep can be routinely performed (UK Legislation 2007). It is stipulated that a rubber ring may only be used on animals younger than seven days of age and any other method requires anaesthesia.

In theory, the presence of the tail would increase the accumulation of faeces and urine in the wool of the perineal region of sheep, which would be detrimental both to the health of the individual in question and the overall performance of the flock (Pugh & Baird 2012). This loss would be directly related to decreased wool quality in the posterior region of the sheep and, indirectly, by difficulties incurred during copulation or an increased incidence of myiasis and infections (Soriano *et al.* 2020). The principle argument in defence of this practice is the supposed positive effect on the health of animals that result from tail removal, especially as regards prevention of myiasis or flystrike; a serious parasitic pathology that affects sheep caused by blowfly larvae (French *et al.* 1994). This belief remains highly prevalent in sheep farming (Larrondo *et al.* 2018; Stamm *et al*. 2019; Woodruff *et al.* 2020). Scientific evidence illustrating the benefit of tail docking in terms of preventing myiasis in sheep is limited and inconclusive. Of the three experimental studies carried out in Australia, only one found a reduced risk of myiasis in docked compared to undocked sheep: while French *et al.* (1994) found a higher incidence of blowfly strike in undocked lambs, this result was not consistent with the findings of Riches (1942) and Ware *et al.* (2000). Sutherland and Tucker (2011) point out in their extensive literature review that additional research is needed to justify the maintenance of tail docking in sheep, especially due to the extensive disadvantages of carrying out such a practice in terms of the animals’ health and well-being.

The widespread use of tail docking in the Brazilian sheep industry today may be related to the historical context of importating management practices from the Australian sheep industry. Myiasis is a major animal health challenge for sheep farming in both Australia (Lihou & Wall 2019) and Brazil (Costa-Júnior *et al.* 2019; Barros & Bricarello 2020). The first study available in the scientific literature investigating the relationship between tail docking and the incidence of myiasis in sheep dates back to the 1950s and was carried out in Queensland, Australia (Riches 1942). Australia had already demonstrated a special aptitude for raising sheep, capitalising on extensive native pasture reserves and natural resources. Much of Brazil’s technical expertise (both past and present) regarding sheep farming originates from Australia (de Ávila *et al.* 2013). The myiasis that most affect Brazilian and Australian sheep are classified as ‘cutaneous myiasis’ or ‘traumatic myiasis’, which show similar clinical presentations. However, in Brazil, the main causal agent involved in sheep myiases differs from that seen in Australia (Hall *et al.* 2016). On the Australian continent, it is the *Lucilia cuprina* fly (Wiedemann 1830) which is the main cause of primary myiases in sheep and it can also found in other parts of the world, including Africa and North America (Hall *et al.* 2016). *Chrysomya bezziana* and *C. hominivorax*, the Old World and New World screw-worm flies, respectively, are exotic to Australia and notifiable under state and territory legislation. All suspected cases should be reported to the relevant state or territory government animal health authority (Australian Government 2023). In Brazil, *C. hominivorax* is the main aetiologic agent of animal myiasis (Costa-Júnior *et al.* 2019) and is endemic in South America (Altuna *et al.* 2021). The larvae of this blowfly have an obligatory biontophagous feeding habit, feeding on the living tissues of warm-blooded animals. Female flies are attracted to the blood of wounds, such as those caused by tail docking in sheep. In these wounds, flies deposit their eggs, and from these eggs larvae will emerge, causing myiasis (Hall *et al.* 2016). Thus, although *L. cuprina* and *C. hominivorax* both belong to the order of Calliphorideos and share many biological similarities, a key distinguishing feature is that *C. hominivorax* does not lay its eggs in a build-up of faeces or urine in the wool but on open wounds and living tissue, injured or not (Hall *et al.* 2016). This seemingly small detail has a large effect on the dynamics of establishing the pathology in each country. Tail docking has been performed for over 50 years in Australia as an alternative to control myiases caused by *L. cuprina*, based on a biological aspect of the fly, which is renowned for its peculiar ability to lay its eggs directly onto wet and dirty wool (Vartib-Browne 1958). The direct transposition of the idea of amputating the sheep’s tail to the Brazilian reality ignores the fact that the main fly causing myiasis in animals and humans is the *C. hominivorax* (Coquerel 1858; Costa-Júnior *et al.* 2019). There is no up-to-date information on the occurrence of sheep myiasis immediately following tail docking in lambs reared in Brazil.

This study evaluated the impact of not tail docking on the incidence of myiasis in an experimental Brazilian flock of a retrospective cohort of six consecutive years. The study aimed to evaluate the effectiveness of tail docking as a protective measure against *C. hominivorax* myiasis and, thus, provide scientific data that can be used to clarify the effectiveness or otherwise of tail docking in sheep in Brazil.

## Materials and methods

### Ethical approval

All the processes and standards that guided the execution of this research are in accordance with the guidelines of the National Council for the Control of Animal Experimentation in Brazil and the Directive 2010/63/EU. The ARRIVE guidelines were adhered to. No animals were euthanased in this study. The research project that gave rise to this study was submitted and approved in the evaluation by the Ethics Commission on Animal Use of the Federal University of Santa Catarina (CEUA-UFSC) under n 6324250619.

### Study animals

The animals used in the study belonged to an experimental flock residing in the Research and Extension Centre in Agroecology of the Experimental Farm of the Federal University of Santa Catarina, in Florianópolis, State of Santa Catarina, Brazil (27º 41’ 06.28” S; 48º32’ 38.81” O).

The flock has been raised at this site from 2014 onwards and during the study period had an average size of approximately 60 animals of different ages (0 to 72 months) consisting of Texel (n = 38), Crioula Lanada (n = 91), Romney Marsh (n = 10), Polwarth (n = 5) breeds and their crosses (n = 63). A total 207 sheep were part of this flock during the study period. The animals born here were not tailed docked, but those originating from other farms that became part of the experimental flock were adult animals already tail docked. Thus, the flock was composed of adult animals both with and without tails and lambs with tails; thus any conclusions drawn regarding the effect of tail docking in myiasis incidence is purely valid for adult animals. A more detailed description of the characteristics of the experimental flock during the study period is shown in Tables 1 and 2.

**Table 1.** Number of recordings (animal-month) made according to year and age within the docked and undocked groups that comprised the data used in the statistical analytical model (n = 4,318)

	Docked			Undocked		
Age (months)	0–3	4–12	>12	0–3	4–12	>12
Year						
2015	0	0	492	182	321	30
2016	0	0	254	26	138	262
2017	0	2	171	96	143	153
2018	0	16	193	92	146	244
2019	0	0	162	84	103	327
2020	0	0	159	39	99	384

**Table 2.** Number of recordings (animal-month) made according to year and breed within the docked and undocked groups that comprised the data used in the statistical analytical model (n = 4,138)

Breed	Crioula Lanada		Texel		Romney March		Polwarth		Crossbreed	
Docked	Yes	No	Yes	No	Yes	No	Yes	No	Yes	No
Year										
2015	252	340	240	183	0	0	0	0	0	10
2016	164	301	90	90	0	0	0	0	0	35
2017	104	253	60	1	9	0	0	0	0	138
2018	45	329	56	0	108	3	0	0	0	150
2019	12	255	42	0	108	2	0	0	0	257
2020	12	190	24	0	108	0	15	0	0	332

The ewes were reared in an agroecological system integrated to the production of vegetables, fruits, and grains. The grazing system in place was Rotational Voisin (Pinheiro Machado 2010) with animals subsisting on improved native pasture and supplementation with special commercial feed for sheep or alfalfa hay up to 1.5% of live weight. Shelter, shade, drinking water and special mineral salt were all freely available.

### Myiasis measures

The diagnosis of myiasis was made by the team of researchers from the Agroecology Centre at Fazenda Ressacada during the daily inspection of the sheep flock. Diagnoses were always carried out by a veterinarian trained in veterinary entomology and confirmation of the species causing myiasis was made via taxonomic identification by the Animal Parasitology Laboratory of the Federal University of Santa Catarina.

### Study design

This is an epidemiological study of the retrospective cohort type in an experimental flock of sheep over a time-frame of six years (2015 to 2020), where the outcome of interest was the monthly incidence of myiasis, and the main predisposing factor studied was the effect of the presence or absence of the tail on animals. The checklist for epidemiological studies in veterinary medicine (STROBE-VET) was used in the construction of this study and is available in the Supplementary material.

The time unit adopted was the month and the experimental unit was each sheep in the flock. The incidence was calculated considering the occurrence of cases of myiasis as a dichotomous variable, thereby generating two possibilities for categorisation: animal with myiasis or animal without myiasis in each month. The classic actuarial method was used to calculate the incidence rates, weighted by the number of individuals at risk in the corresponding month. This choice aimed to control the effect of entry and exit of animals from the flock during the study period. Thus, the values for incidence are presented in percentage rates that are the result of the absolute division of the ‘number of sheep affected by myiasis in the month’ by the ‘number of sheep in the flock in the same month’.

Here, the incidence rate of myiasis was the outcome of interest. Relative risk (RR) and odds ratio (OR) were the association measures used in this study. The incidence rate ratio (IRR) was used as an aid to evaluate the effectiveness of tail docking to reduce the incidence of myiasis. The differences verified on the association measures provide the empirical basis for the discussion.

### Statistical analysis

The primary data were obtained from field records and organised with the support of the Microsoft Excel® software version 2019. All statistical analyses were performed with the support of software IBM-SPSS® version 25.0. Descriptive statistics were initially used to present the information obtained after the integration of the data sets according to the analysed predictors. The RR was calculated from contingency tables submitted to the statistical model of Poisson regression. The Chi-squared test was used to show statistical differences between groups. The OR were calculated by binary logistic regression. The IRR was calculated by Poisson Regression with robust estimation of variance. The confidence interval adopted was always 95% (*P* < 0.05).

## Results

All information presented here is available in a public and free manner accessible by the following link in Dataset Mendeley®: https://data.mendeley.com/datasets/xk9zrx6t9r/1. All raw data and the statistically analysed data that originated from this study are stored in this digital repository.

The cohort lasted 72 months (January 2015 to December 2020) and during this period the flock was composed of a mean (± SEM) number of 59.79 (±14.36) animals monthly. The outcomes of all animals (n = 207) that made up the flock during the cohort were monitored, totalling 4,318 entries (recordings per animal-month) that supplied the base statistical analytical model. Our study found a concentration of myiasis in sheep without tails. Animals without tails presented 66.4% (n = 2,869) of the total myiases that occurred in the flock during the period studied, while sheep with tails were affected by 33.6% (n = 1,449) of myiases.

All myiasis reported here were caused by *C. hominivorax*, as confirmed by laboratory diagnosis carried out by a specialist veterinarian.

### Effect of presence of an intact tail on the occurrence of *C. hominivorax* myiasis

Table 3 presents information regarding the incidence of *C. hominivorax* myiasis in the flock, in terms of its distribution according to the presence or absence of the tail (i.e. undocked or docked) in animals that were affected or not by myiasis during the study period.

**Table 3.** Incidence rate of myiasis according to the tail-docking status (docked, undocked) of the sheep monitored. Percentage values with different letters in the same column differ significantly (Chi-squared test; *P* < 0.001)

			**Myasis recorded**		
			Yes	No	Total
Tail	Docked	(n)	126	1,323	1,149
		(%)	8.7^a^	91.3^a^	100
	Undocked	(n)	114	2,755	2,869
		(%)	4.0^b^	96.0^b^	100
Total		(n)	240	4,078	4,318
		(%)	5.6	94.4	100

The results indicate the association between the occurrence of myiasis in sheep and the outcome variable categorised as ‘Tail’, with two categories: ‘Undocked’ and ‘Docked’. The ‘Undocked’ category was used as the reference for comparison. For the undocked tail group, set as the reference, the results are presented as a basis for comparison with the docked tail group. The findings reveal that the relative risk of myiasis occurrence in sheep with docked tails is significantly higher, with a value of 2.1 (95% CI: 1.7–2.7) compared to the undocked tail group. This finding is supported by an odds ratio of 2.3 (95% CI: 1.7–2.9), indicating a greater likelihood of developing myiasis in the docked tail group. Furthermore, the incidence rate ratio for the docked group is a value of 0.7 (95% CI: 0.5–1.0). The incidence rate ratio was calculated using the number of docked animals as a reference. All *P*-values associated with these measures are significantly low (*P* < 0.001), indicating that these associations are statistically significant. The 95% confidence interval analyses reinforce these results, providing a range of values within which the true effect is likely to lie.

## Discussion

Tail docking did not decrease the risk of sheep being affected by myiasis by *C. hominivorax.* Furthermore, the analysis of the IRR of myiasis between the docked and undocked sheep showed that the caudectomy was ineffective in protecting the animals from myiasis. Other Brazilian studies have also shown that tail docking did not prevent the occurrence of myiasis in sheep (Madeira *et al.* 1998; Stamm *et al*. 2019). Moreover, we found a significant positive association between tail docking and the occurrence of myiasis, indicating that docking may be a risk factor for myiasis by *C. hominivorax* in sheep. The combination of these results supports the hypothesis that tail docking is not a protective factor against myiasis in sheep. Our results contribute to a growing list of other studies that call for a rethinking of tail docking of sheep as a blanket measure to prevent myiasis in Brazil.

Ratio-based measures of association, such as RR, OR, and IRR, provide solid information on the strength of association between the factors studied (whether risk or protection) and the outcome of interest. These measures are part of a critical group of metrics recognised as the ‘gold standard’ in the study of possible disease determinants, especially in cohort studies (White *et al.* 1998). Due to the lack of docked lambs in the sample, as reported in the methodology, this bias must be considered. The sampling process used in this study gains strength due to the extensive period that was analysed.

Other studies have also found greater rates of occurrence of myiasis in docked compared to undocked sheep in both Brazil (Madeira *et al.* 1998) and Australia (Riches 1942). Although this evidence may suggest that we should consider tail docking as a risk factor for the occurrence of *C. hominivorax* myiasis in sheep in Brazil, it is necessary to be cautious when making this extrapolation, as this type of causal relationship can only be inferred by the integration of large amounts of repeated data in different times and conditions (Ahlbom 1984). Thus, further longitudinal epidemiological studies are needed to better understand this relationship. However this association may be partially explained, through a consideration of the basic function of the sheep’s tail, notably as a swatting appendage, warding off flies and other arthropods from the posterior region. This trait, also present in cattle and horses, played a key role in the successful evolution of these species (Hickman 1979). The ‘fly-swatter’ type of movements performed by the tail is part of a group of typical behaviours that are evolutionarily fixed in most ruminant species and confer a protective function against attacks by flies (Mooring *et al.* 2007). and perhaps explain why a concentration of incidence of myiasis in sheep submitted to tail docking was observed in this study and in that of Madeira *et al.* (1998). The posterior region (mainly the perineum, anus and vulva region) has been one of the most affected anatomical sites for myiasis in studies that sought to map distribution over the body of sheep (Snoep *et al.* 2002; Sotiraki & Hall 2012).

The lack of scientific support makes it very difficult for public and private agents with an effective capacity to carry out actions and changes related to the welfare of farm animals to make decisions (Fraser 2008). This challenge is especially critical in the context of management practices that are already strongly inserted into the ‘ethos’ of productive activity, such as tail docking in sheep farming. By turning to these contemporary demands, academic research can contribute to the ethical advancement of society. We hope that the results of this study can add substantially to the public debate on sheep tail docking. Importantly, changing the practice also requires a consideration of the cultural aspects of the sheep industry. Farmers believe tail docking to be painful yet necessary and use it in their farms (Larrondo *et al.* 2018; Stamm *et al.* 2019). In Brazil, tail docking is recommended by most training courses for field technicians and sheep farmers, and this recommendation also appears in most technical publications on sheep farming (Stamm *et al.* 2019; Soriano *et al.* 2020). Stamm *et al*. (2019) reported that the vast majority of South Brazilian sheep farmers believe tail docking to be essential for the success of sheep farming as a whole and have no intentions of abandoning this practice.

### Animal welfare implications

This study demonstrated that tail docking is not a protective factor against the occurrence of myiasis in sheep and that, on the contrary, it may represent a risk factor in some cases. These findings contribute to the evidence-based arguments regarding the need for tail docking in sheep, especially in the context of Brazil and Latin America. The information presented here may prove useful for sheep farmers when deciding whether or not to dock the tails of their animals.

## Conclusion

Tail docking did not decrease the risk of *C. hominivorax* myiasis in the sheep monitored in this study but increased the odds of occurrence of this disease during the period analysed. Our findings suggest that the argument used to justify tail docking, namely that it would benefit the health of the animals by preventing flystrike, may not be supported by the evidence.

## Supporting information

Barros et al. supplementary material Barros et al. supplementary material

## Supplementary material

The supplementary material for this article can be found at http://doi.org/10.1017/awf.2024.21.

## References

[r1] Ahlbom A 1984 Criteria of causal association in epidemiology. Health, Disease, and Causal Explanations in Medicine (3): 93–98. 10.1007/978-94-009-6283-5_12

[r2] Alonso ME, González-Montaña JR and Lomillos JM 2020 Consumers’ concerns and perceptions of farm animal welfare. Animals 10(3): 385. 10.3390/ANI10030385 32120935 PMC7143148

[r3] Altuna M, Hickner PV, Castro G, Mirazo S, Pérez de León AA and Arp AP 2021 New World screwworm (*Cochliomyia hominivorax*) myiasis in feral swine of Uruguay: One Health and transboundary disease implications. Parasites and Vectors 14(26). 10.1186/s13071-020-04499-z PMC7789611 33413607

[r4] Australian Government 2023 *Exotic Animal Diseases Bulletin - No.* 109. https://www.agriculture.gov.au/biosecurity-trade/pests-diseases-weeds/animal/ead-bulletin/ead-bulletin-109 (accessed 3 April 2024).

[r5] Barros GP de and Bricarello PA 2020 Myiasis by *Cochliomyia hominivorax* (Coquerel, 1858): A neglected zoonosis in Brazil. Open Journal of Veterinary Medicine 10: 80–91. 10.4236/ojvm.2020.106007

[r6] Connor M and Cowan SL 2020 Consumer evaluation of farm animal mutilations. Research in Veterinary Science 128(1): 35–42. 10.1016/j.rvsc.2019.10.006 31707098

[r7] Conselho Federal de Medicina Veterinária 2008 *Normative Resolution no. 877 of February 15, 2008.* https://www.camara.leg.br/proposicoesWeb/prop_mostrarintegra?codteor=585565#:~:text=Disp%C3%B5e%20sobre%20os%20procedimentos%20cir%C3%BArgicos,animais%20e%20d%C3%A1%20outras%20provid%C3%AAncias (accessed 13 March 2024).

[r8] Conselho Federal de Medicina Veterinária 2009 *Normative Resolution No. 928 of December 13, 2009.* https://www.normasbrasil.com.br/norma/resolucao-928-2009_110262.html#:~:text=Fica%20proibida%20a%20realiza%C3%A7%C3%A3o%20de,o%20comportamento%20natural%20da%20esp%C3%A9cie.%22 (accessed 13 March 2024)

[r9] Coquerel C 1858 Note on larvae belonging to a new species of diptera (*Lucilia hominivorax*). Annales Societe Entomologique de France 27: 171–176.

[r10] Costa-Júnior LM, Chaves DP, Brito DRB, Santos VAF dos, Costa-Júnior HN and Barros ATM 2019 A review on the occurrence of *Cochliomyia hominivorax* (Diptera: Calliphoridae) in Brazil. Revista Brasileira de Parasitologia Veterinária 28(4): 548–562. 10.1590/s1984-29612019059 31483031

[r11] de Ávila VS, Fruet APB, Barbieri M, Bianchini NH and Dörr AC 2013 The return of sheep farming to the production scenario of Rio Grande Do Sul. Revista Eletrônica em Gestão, Educação e Tecnologia Ambiental 11(11): 2419–2426.

[r12] Fraser D 2008 Understanding animal welfare. Acta Veterinaria Scandinavica 50(S1): 1–7. 10.1186/1751-0147-50-S1-S1 19049678 PMC4235121

[r13] French NP, Wall R and Morgan KL 1994 Lamb tail docking: a controlled field study of the effects of tail amputation on health and productivity. The Veterinary record 134: 463–467. 10.1136/vr.134.18.463 8059511

[r14] Hall MJR, Wall RL and Stevens JR 2016 Traumatic myiasis: A neglected disease in a changing world. Annual Review of Entomology 61: 159–176. 10.1146/annurev-ento-010715-023655 26667275

[r15] Hickman GC 1979 The mammalian tail: a review of functions. Mammal Review 9(4): 143–157. 10.1111/j.1365-2907.1979.tb00252.x

[r16] Hötzel MJ and Vandresen B 2022 Brazilians’ attitudes to meat consumption and production : Present and future challenges to the sustainability of the meat industry. Meat Science 192: 108893. 10.1016/j.meatsci.2022.108893 35760024

[r17] Instituto Brasileiro de Geografia e Estat í stica - IBGE 2021 *Herd of sheep (sheep and rams).* https://www.ibge.gov.br/explica/producao-agropecuaria/ovino/br (accessed 3 April 2024).

[r18] Larrondo C, Bustamante H and Gallo C 2018 Sheep farmers’ perception of welfare and pain associated with routine husbandry practices in Chile. Animals 8(12): 1–14. 10.3390/ani8120225 PMC6315487 30487400

[r19] Lihou K and Wall R 2019 Sheep blowfly strike: The cost of control in relation to risk. Animal 13(10): 2373–2378. 10.1017/S1751731119000831 31062673

[r20] Madeira NG, Amarante AFT and Padovani CR 1998 Effect of management practices on screw-worm among sheep in São Paulo State, Brazil. *Tropical Animal Health and Production* 30: 149–157. 10.1023/A:1005055518916 9719843

[r21] Minist é rio da Agricultura, Pecu á ria e Abastecimento - MAPA 2021 *Ordinance of the Ministry of Agriculture, Livestock and Supply no.* 52*, of March 15, 2021.* https://www.gov.br/agricultura/pt-br/assuntos/sustentabilidade/organicos/arquivos-organicos/PORTARIA_MAPA_N_52.2021_ALTERADA_PELA_PORTARIA_MAPA_N_404.pdf (accessed 13 March 2024).

[r22] Mooring MS, Blumstein DT, Reisig DD, Osborne ER and Niemeyer JM 2007 Insect-repelling behaviour in bovids: Role of mass, tail length, and group size. Biological Journal of the Linnean Society 91(3): 383–392. 10.1111/j.1095-8312.2007.00803.x

[r23] Orihuela A and Ungerfeld R 2019 Tail docking in sheep (*Ovis aries*): A review on the arguments for and against the procedure, advantages/disadvantages, methods, and new evidence to revisit the topic. Livestock Science 230: 103837. 10.1016/j.livsci.2019.103837

[r24] Pinheiro Machado LC 2010 Voisin Rational Grazing: Agroecological Technology for the Third Millennium, Third *Edition* pp 373. Expressão Popular: São Paulo, Brazil.

[r25] Pugh DG and Baird AN 2012 Sheep and Goat Medicine, Second Edition. Elsevier Inc: Maryland Heights, MO, USA. 10.1016/C2009-0-60474-8

[r26] Riches JH 1942 Further observations on the relation of tail length to the incidence of blowfly strike of the breech of Merino sheep. Journal of the Council for Scientific and Industrial Research, Australia 15(1): 3–9. https://www.cabidigitallibrary.org/doi/full/10.5555/19431000067 (accessed 3 April 2024).

[r27] Sheep Standards and Guidelines 2013 Sheep standards and guidelines – Tail docking discussion paper. *Sheep tail docking discussion paper public consultation version* 1.3.13: 1–17. https://www.animalwelfarestandards.net.au/files/2011/05/Sheep-Tail-docking-discussion-paper-5.3.13.pdf (accessed 13 March 2024).

[r28] Snoep JJ, Sol J, Sampimon OC, Roeters N, Elbers ARW, Scholten HW and Borgsteede FHM 2002 Myiasis in sheep in The Netherlands. Veterinary Parasitology 106(4): 357–363. 10.1016/S0304-4017(02)00088-2 12079741

[r29] Soriano VS, Phillips CJC, Taconeli CA, Fragoso AAH and Molento CFM 2021 Mind the gap: Animal protection law and opinion of sheep farmers and lay citizens regarding animal maltreatment in sheep farming in southern brazil. Animals 11(7): 1903. 10.3390/ani11071903 34206770 PMC8300268

[r30] Soriano VS, Stamm FO, Taconeli CA and Molento CFM 2020 To dock or not to dock? Faecal soiling measurement in sheep. Animal Welfare 29(1): 81–87. 10.7120/09627286.29.1.081

[r31] Sotiraki S and Hall MJR 2012 A review of comparative aspects of myiasis in goats and sheep in Europe. Small Ruminant Research 103(1): 75–83. 10.1016/j.smallrumres.2011.10.021

[r32] Stamm F de O, Molento MB and Molento CFM 2019 South Brazilian farmers’ perceptions concerning sheep tail docking. Ciencia Rural 49(4): 1–6. 10.1590/0103-8478cr20180571

[r33] Sutherland MA and Tucker CB 2011 The long and short of it: A review of tail docking in farm animals. Applied Animal Behaviour Science 135(3): 179–191. 10.1016/j.applanim.2011.10.015

[r34] UK Legislation 2007 *The Mutilations (Permitted Procedures) (England and Wales) Regulations 2007.* https://www.legislation.gov.uk/uksi/2007/1100 and https://www.legislation.gov.uk/wsi/2007/1029 (accessed 11 March 2024).

[r35] Vargas-Bello-Pérez E, Miranda-de la Lama GC, Teixeira DL, Enríquez-Hidalgo D, Tadich T and Lensink J 2017 Farm animal welfare influences on markets and consumer attitudes in Latin America: The cases of Mexico, Chile and Brazil. *Journal of Agricultural and Environmental Ethics* 30: 697–713. 10.1007/s10806-017-9695-2

[r36] Vartib-Browne L 1958 The choice of communal Oviposition sites by the Australian sheep blowfly. *Lucilia cuprina.* *Australian Journal of Zoology* 6(3): 241–247. 10.1071/ZO9580241

[r37] Ware JKW, Vizard AL and Lean GR 2000 Effects of tail amputation and treatment with an albendazole controlled-release capsule on the health and productivity of prime lambs. Australian Veterinary Journal 78(12): 838–842. 10.1111/j.1751-0813.2000.tb10504.x 11194472

[r38] White E, Hunt JR and Casso D 1998 Exposure measurement in cohort studies: The challenges of prospective data collection. Epidemiologic Reviews 20(1): 43–56. 10.1093/oxfordjournals.epirev.a017971 9762508

[r39] Wiedemann C 1830 Non-European two-winged insects. Schulzischen Buchhandlung 1: 38–42.

[r40] Woodruff ME, Doyle R, Coleman G, Hemsworth L and Munoz C 2020 Knowledge and attitudes are important factors in farmers’ choice of lamb tail docking length. Veterinary Record 186(10): 319. 10.1136/vr.105631 31959706

