# Supplementary material for: Does tail docking prevent Cochliomyia hominivorax myiasis in sheep? A six-year retrospective cohort study
Source: Anim Welf. 2024 May 8;33:e26. doi: 10.1017/awf.2024.21 (PMC11094548; doi:10.1017/awf.2024.21)
Supplement: Barros et al. supplementary material [file S0962728624000216sup001.pdf]

The STROBE-Vet statement checklist.

	Item	*STROBE-Vet recommendation*	*Section#*
**Title and Abstract**	1	(a) Indicate that the study was an observational study and, if applicable, use a common study design term	The title already states the nature of the study and uses the common term.
		(b) Indicate why the study was conducted, the design, the results, the limitations, and the relevance of the findings	The abstract informs these points
**Background / rationale**	2	Explain the scientific background and rationale for the investigation being reported	The introduction informs these points
**Objectives**	3	(a) State specific objectives, including any primary or secondary prespecified hypotheses or their absence	The introduction informs these points
		(b) Ensure that the level of organization is clear for each objective and hypothesis	The introduction informs these points
**Study design**	4	Present key elements of study design early in the paper	The introduction informs these points

**Setting**	5	(a) Describe the setting, locations, and relevant dates, including periods of recruitment, exposure, follow-up, and data collection	The methodology informs these points
		(b) If applicable, include information at each level of organization	The methodology informs these points
**Participants** [b]	6	(a) Describe the eligibility criteria for the owners/managers and for the animals, at each relevant level of organization	The methodology informs these points
		(b) Describe the sources and methods of selection for the owners/managers and for the animals, at each relevant level of organization	The methodology informs these points
		(c) Describe the method of follow-up	The methodology informs these points
		(d) For matched studies, describe matching criteria and the number of matched individuals per subject (e.g., number of controls per case)	Not applicable in this study
**Variables**	7	(a) Clearly define all outcomes, exposures, predictors, potential confounders, and effect modifiers. If applicable, give diagnostic criteria	The methodology informs these points
		(b) Describe the level of organization at which each variable was measured	The methodology informs these points
		(c) For hypothesis-driven studies, the putative causal-structure among variables should be described (a diagram is strongly encouraged)	No diagrams were used,

			but the theoretical hypothesis was clearly described in the introduction.
**Data sources / measurement**	8*	(a) For each variable of interest, give sources of data and details of methods of assessment (measurement). If applicable, describe comparability of assessment methods among groups and over time	The methodology informs these points
		(b) If a questionnaire was used to collect data, describe its development, validation, and administration	Not applicable in this study
		(c) Describe whether or not individuals involved in data collection were blinded, when applicable	Not applicable in this study
		(d) Describe any efforts to assess the accuracy of the data (including methods used for “data cleaning” in primary research, or methods used for validating secondary data)	The methodology informs these points
**Bias**	9	Describe any efforts to address potential sources of bias due to confounding, selection, or information bias	The methodology informs these points
**Study size**	10	(a) Describe how the study size was arrived at for each relevant level of organization	The methodology informs these points
		(b) Describe how non-independence of measurements was incorporated into sample-size considerations, if applicable	Not applicable in this study

		(c) If a formal sample-size calculation was used, describe the parameters, assumptions, and methods that were used, including a justification for the effect size selected	The study included all events that occurred during the analysis time.
**Quantitative variables**	11	Explain how quantitative variables were handled in the analyses. If applicable, describe which groupings were chosen, and why	The methodology informs these points
**Statistical methods**	12	(a) Describe all statistical methods for each objective, at a level of detail sufficient for a knowledgeable reader to replicate the methods. Include a description of the approaches to variable selection, control of confounding, and methods used to control for non-independence of observations	The methodology informs these points
		(b) Describe the rationale for examining subgroups and interactions and the methods used	Not applicable in this study
		(c) Explain how missing data were addressed	The methodology informs these points
		(d) If applicable, describe the analytical approach to loss to follow-up, matching, complex sampling, and multiplicity of analyses	The methodology informs these points
		(e) Describe any methods used to assess the robustness of the analyses (e.g., sensitivity analyses or quantitative bias assessment)	The methodology informs these points

**Participants**	13*	(a) Report the numbers of owners/managers and animals at each stage of study and at each relevant level of organization - e.g., numbers eligible, included in the study, completing follow-up, and analyzed	The methodology informs these points
		(b) Give reasons for non-participation at each stage and at each relevant level of organization	The methodology informs these points
		(c) Consider use of a flow diagram and/or a diagram of the organizational structure	No flow diagrams were used, but the organization al structure was clearly described in the methodology
**Descriptive data on exposures and potential confounders**	14*	(a) Give characteristics of study participants (e.g., demographic, clinical, social) and information on exposures and potential confounders by group and level of organization, if applicable	The methodology informs these points
		(b) Indicate number of participants with missing data for each variable of interest and at all relevant levels of organization	The methodology informs these points
		(c) Summarize follow-up time (e.g., average and total amount), if appropriate to the study design	The methodology informs these points
**Outcome data**	15*	(a) Report outcomes as appropriate for the study design and summarize at all relevant levels of organization	The results inform these points

		(b) For proportions and rates, report the numerator and denominator	The results inform these points
		(c) For continuous outcomes, report the number of observations and a measure of variability	The results inform these points
**Main results**	16	(a) Give unadjusted estimates and, if applicable, adjusted estimates and their precision (e.g., 95% confidence interval). Make clear which confounders and interactions were adjusted. Report all relevant parameters that were part of the model	Not applicable in this study
		(b) Report category boundaries when continuous variables were categorized	Not applicable in this study
		(c) If relevant, consider translating estimates of relative risk into absolute risk for a meaningful time period	The results inform these points
**Other analyses**	17	Report other analyses done,-such as sensitivity/robustness analysis and analysis of subgroups	Not applicable in this study
**Key results**	18	Summarize key results with reference to study objectives	The results inform these points
**Strengths and Limitations**	19	Discuss strengths and limitations of the study, taking into account sources of potential bias or imprecision. Discuss both direction and magnitude of any potential bias	The discussion informs these points
**Interpretation**	20	Give a cautious overall interpretation of results considering objectives, limitations, multiplicity of analyses, results from similar studies, and other relevant evidence	The discussion informs these points
**Generalizability**	21	Discuss the generalizability (external validity) of the study results	The discussion informs these points

~~**Funding**~~ **<u>Transparency</u>**	22	(a) Funding- Give the source of funding and the role of the funders for the present study and, if applicable, for the original study on which the present article is based<br>(b) Conflicts of interest-Describe any conflicts of interest, or lack thereof, for each author<br>(c) Describe the authors' roles- Provision of an authors' declaration of transparency is recommended<br>(d) Ethical approval- Include information on ethical approval for use of animal and human subjects<br>(e) Quality standards-Describe any quality standards used in the conduct of the research	All these topics appear in the manuscript in their respective sections.

[a] Level of organization recognizes that observational studies in veterinary research often deal with repeated measures (within an animal or herd) or animals that are maintained in groups (such as pens and herds); thus, the observations are not statistically independent. This non-independence has profound implications for the design, analysis, and results of these studies.
[b] The word "participant" is used in the STROBE statement. However, for the veterinary version, it is understood that "participant" should be addressed for both the animal owner/manager and for the animals themselves.
*Give such information separately for cases and controls in case-control studies and, if applicable, for exposed and unexposed groups in cohort and cross-sectional studies.
